# Current Role of Lipoprotein Apheresis in the Treatment of High-Risk Patients

**DOI:** 10.3390/jcdd5020027

**Published:** 2018-05-09

**Authors:** Ulrich Julius

**Affiliations:** Lipidology and Center for Extracorporeal Treatment, Department of Internal Medicine III, University Hospital Carl Gustav Carus at the Technische Universität Dresden, Fetscherstr. 74, 01307 Dresden, Germany; ulrich.julius@uniklinikum-dresden.de; Tel.: +49-351-458-2306; Fax: +49-351-458-5306

**Keywords:** LDL-cholesterol, lipoprotein(a), triglycerides, cardiovascular events, lipoprotein apheresis, PCSK9 inhibitors, lomitapide, antisense oligonucleotide against apolipoprotein(a)

## Abstract

Lipoprotein apheresis (LA) is a therapeutic approach to save the lives of patients who are at an extremely high risk of developing cardiovascular events (CVE), especially after all other therapeutic options were not tolerated, or appeared not to be effective enough. Homozygous familial hypercholesterolemia represents a clear indication to start LA therapy. Another recognized indication is a severe hypercholesterolemia, which induced CVE, often in association with other risk factors. In the last years, an expressive elevation of lipoprotein(a) (Lp(a)) emerged as an indication for LA. In Germany, progress of atherosclerosis should have been documented before the permission to start LA therapy is given in these patients. Usually, all LA methods acutely decrease both LDL-C and Lp(a). However, specific columns which reduce only Lp(a) are available. Case reports and prospective observations comparing the situation before and during LA therapy clearly show a high efficiency with respect to the reduction of CVE, especially in patients with high Lp(a) levels. PCSK9 inhibitors may reduce the need for LA in patients with heterozygous or polygenetic hypercholesterolemia, but in some patients, a combination of these drugs with LA will be necessary. In the future, an antisense oligonucleotide against apolipoprotein(a) may offer an alternative therapeutic approach.

## 1. Introduction

In a recent publication, the author described the history of research in atherosclerosis, of the introduction of lipid-lowering drugs, and of lipoprotein apheresis (LA) into medical practice [1]. In fact, a real breakthrough with respect to cardiovascular outcome data occurred only in the 1990s, with the use of statins, which nowadays represent the basic therapy in patients with hypercholesterolemia (HCH), when tolerated. For ezetimibe, an endpoint study was published only in 2015 [2]. On the other hand, patients with homozygous familial HCH were characterized by an extremely high mortality. Thus, an extracorporeal therapy was at first started in these patients. Initially, a plasma exchange was performed, which has been replaced in the following years by more specific methods. Another indication which was then officially recognized for an extracorporeal therapy appeared to be a severe HCH associated with elevated LDL-cholesterol (LDL-C) concentrations, associated with cardiovascular diseases, despite the application of effective lipid-lowering drugs. In the last years, an elevation of lipoprotein(a) (Lp(a)) played an increasing role among those patients starting LA. This is explained by the more and more generally accepted significance of this atherogenic risk factor, and the absence of any effective drug to reduce Lp(a) levels and cardiovascular endpoints.

This review focuses on the current role of LA in patients with lipid disorders, who in the vast majority, suffer from severe and life-threatening cardiovascular diseases, like myocardial infarction, peripheral arterial occlusive disease, occlusion of the carotids, stroke, atherosclerotic lesions at the aorta, and stenosis of the aortic valve. Of course, the rules governing in Germany are the focus. In daily practice, the term “lipid apheresis” is often being used. However, in fact, this term is not correct—lipids are transported in the blood with complex particles, the lipoproteins. And the extracorporeal treatment removes lipoproteins from the blood—that is why “lipoprotein apheresis” should be preferred.

## 2. Homozygous Familial HCH

The prognosis of patients with homozygous familial HCH is rather poor—without any treatment, they will die at the age of 20 years, or even earlier. After starting an extracorporeal therapy, this life span can be essentially prolonged [3,4]. This has been shown for plasma exchange, as well as for the more specific LA. In the long run, plasma exchange is disadvantageous. Usually, all lipid-lowering drugs (statins, ezetimibe, even PCSK9 inhibitors) are less effective in these patients, compared with other patients. In some of them with absent LDL-receptor function, PCSK9 inhibitors appeared to be totally ineffective.

In fact, LA can acutely reduce LDL-C levels, but due to the rather high pre-LA LDL-C concentrations, target values suggested in international guidelines will hardly be reached. That means that these patients will develop cardiovascular complications (e.g., coronary heart disease, stenosis of the aortic valve), even when treated with LA.

Homozygous familial HCH is internationally recognized as an indication for LA. In Germany, about 100 patients are regularly treated with LA. In other countries, this number is much lower. Even in the Netherlands, where screening programs for familial HCH have been carried out on a large scale, only a few patients are effectively treated with LA.

## 3. Severe Hypercholesterolemia (HCH)

**Figure 1:**
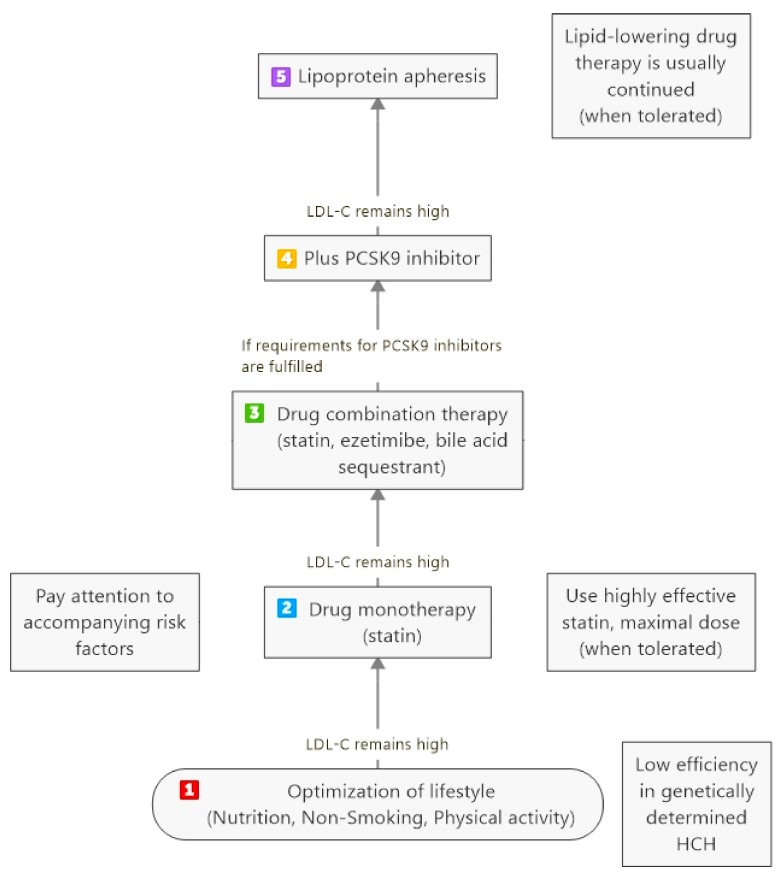
Steps to treat severe hypercholesterolemia (HCH)—from lifestyle to lipoprotein apheresis (LA).

Severe HCH is defined by increased LDL-C levels which have induced atherosclerotic lesions in different vessels. An optimal diet should be adhered to—though its effectiveness with respect to lowering LDL-C levels in patients with a genetically determined disease (mutations at the LDL-receptor gene, at the PCSK9 gene, at the apolipoprotein B gene) is rather limited. In all patients with cardiovascular complications, drug therapy (statins when tolerated, ezetimibe, bile acid sequestrants) is always needed. Whereas in patients with polygenetic HCH the LDL-C target level for high-risk patients (1.8 mmol/L) can often be reached, in those with genetically caused HCH, this is usually not the case. In Germany, the Joint Federal Committee (which is in charge of registration of new therapeutic approaches) demands that the lifestyle/drug therapy should be performed for at least 1 year before LA treatment can be started [5]. This is a rather long time for patients with extremely high risk. Moreover, it has to be taken into account that additional risk factors (diabetes, hypertension, elevated triglycerides, low HDL-cholesterol, renal insufficiency, positive family history for early cardiovascular events) contribute to this cardiovascular risk. The authorities did not define a target level for LDL-C for starting LA treatment, evidently, international guidelines are accepted. However, the cardiovascular risk due to all risk factors in a given patient has to be taken into consideration when thinking about LA therapy. Clearly, LA therapy is the last step in the therapy of HCH (Figure 1).

In general, the LA indication is accepted within secondary prevention, that is, in order to prevent new cardiovascular events. In exceptional cases, with extremely elevated LDL-C concentrations despite an effective and tolerated drug therapy, LA may be started in patients who did not yet suffer from cardiovascular events. Usually these patients have a family background of cardiovascular diseases, and show early atherosclerotic plaques at some vessels. 

In Germany, in order to get the permission for LA, an application has to be written which should be accompanied by a lipidologic evaluation and should be approved by apheresis committees at the regional Associations of Statutory Health Care Physicians. Only nephrologists are allowed to treat patients with LA.

In the last years, the number of patients starting LA treatment in Germany with the diagnosis “severe hypercholesterolemia” amounted to about 150 per year.

Following an LA session, LDL-C concentrations increase and reach pre-LA levels approximately after one week. At least in Germany, the general recommendation is that patients should be treated with LA once per week.

## 4. Isolated Elevation of Lp(a)

Since 2000, an elevation of Lp(a) was increasingly recognized as an atherogenic risk factor. Some patients got the permission to be treated with LA for this indication via legal proceedings. In 2008, the Joint Federal Committee decided to accept an isolated elevation of Lp(a) as an official indication for LA [6], though the first study reporting the effects of LA on cardiovascular endpoints was only published in 2009. In other countries, this indication is only partially recognized (Great Britain, Italy). Danish scientists contributed a lot with respect to the characterization of Lp(a) as a risk factor—LA does not play any role in Denmark.

At present, LA is the only available effective therapeutic approach to lower Lp(a). Previously, niacin was demonstrated to lower Lp(a) by about 20%. However, outcome data did not prove any benefit from this reduction [7].

Patients who develop cardiovascular events due to high Lp(a) concentrations typically have the following peculiarities:

1. Lp(a) levels are extremely high.
2. A positive family history with early cardiovascular events in first-degree relatives is often seen.
3. Patients suffer from a first severe cardiovascular event (reanimation needed after an acute myocardial infarction) already at young age (before 50).
4. Many patients underwent several interventions (PTCA, PCI) before the diagnosis of an elevation of Lp(a) is made.
5. Very often they were heavy smokers—the combination of smoking and high Lp(a) is especially atherogenic.
6. Patients have a concomitant hypertension.

The Joint Federal Committee defined two criteria for the initiation of LA in these patients: (1) Lp(a) level higher than 60 mg/dL (or higher than 120 nmol/L when newer lab methods are used); (2) progress of atherosclerotic disease, either clinically or documented with an imaging technique. In the experience of the author, the first criterion was never a problem—though it is higher than the Lp(a) level when atherosclerosis starts to develop (about 30 mg/dL). However, progress is difficult to understand—even the first cardiovascular event in a patient is the result of a progress of an atheroma. The vast majority of patients starting LA treatment with the diagnosis “isolated elevation of Lp(a)” suffered from multiple events, before the treating physician remembered that Lp(a) may play a role. A special problem represents very young patients (under 30 years old) who underwent an acute myocardial infarction which they survived with the help of modern reanimation techniques. A progress would mean another myocardial infarction which probably will be lethal. The apheresis committees usually accepted the indication for LA in these patients.

The treating doctor is required to optimize all other risk factors: (1) LDL-C should be lowered below 1.8 mmol/L—usually statins (and ezetimibe and/or bile acid sequestrant) are administered when tolerated; (2) blood pressure should be normalized—often drugs are needed; (3) in case of overweight, weight should be reduced—dietary advice should be given to all patients, also with respect to a coexisting dyslipidemia; (4) patients should stop smoking; (5) special attention should be paid to diabetes or renal insufficiency.

In Germany, the number of new patients with the diagnosis “isolated elevation of Lp(a)” was increasing in the last years. In 2016, 412 patients (equivalent to 73% of all new patients) have been accepted with this indication for the first time. At our Center for Extracorporeal Therapy, all new patients who came to be treated with LA in 2017 showed elevated Lp(a) concentrations. However, a few among them had an elevation of both Lp(a) and LDL-C, though they were treated with lipid-lowering drugs.

The numbers for other countries are unknown.

In the days after an LA session, Lp(a) levels steadily increase. Thus, it makes sense to treat the patients with LA once per week. In other countries, a biweekly regimen is common. Among our patients, we saw a few whose pre-LA Lp(a) concentrations was reached already after 3 days. In these patients, especially when they suffer from new cardiovascular events (CVE), despite being treated with LA, two LA sessions per week may be advisable.

## 5. Lipoprotein Apheresis—What Is the Evidence

### 5.1. Evidence for the Effects of LA Treatment

**Figure 2:**
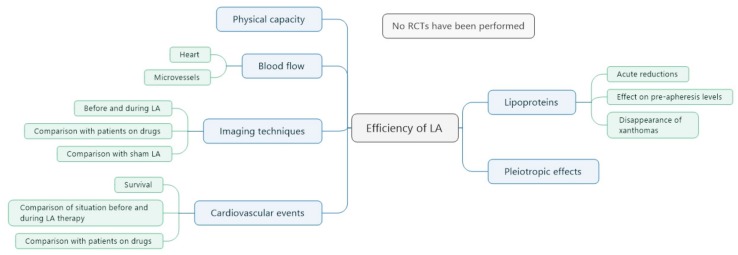
Criteria for evaluation of efficiency of LA. RCTs: randomized controlled studies.

Lipid concentrations and the development of atherosclerosis are major criteria for evaluation of the efficiency of LA therapy (Figure 2).

Usually, LDL-C and Lp(a) levels are regularly measured before and after the LA sessions. In the above-mentioned rules of the Joint Federal Committee, an acute reduction of LDL-C by more than 60% is given as a quality criterion. In the daily practice, this reduction can be easily reached, often, reductions of LDL-C up to 80% are seen. Over the years, pre-apheresis LDL-C levels may be decreased. In patients with familial HCH xanthomas disappear.

With respect to reduction rates for Lp(a) levels, no official rules have been published. Acute reductions of Lp(a) by 70–90% are realistic. When comparing with Lp(a) concentrations, before the start of LA treatment, pre-apheresis Lp(a) are about 20% lower [8,9]. In contrast to LDL-C, these pre-apheresis Lp(a) concentrations may even increase in the long run [9].

For LA, no randomized controlled long-term study had been performed. In 2008, the Joint Federal Committee requested to start such a study for patients with elevated Lp(a) levels, but the ethics committee did not approve such a study design.

In a study of the incidence of coronary events in apheresis patients, compared with patients being treating with statins alone (all patients had HCH), the rate was found to be reduced by 72% over 6 years [10]. Several observational studies saw a reduction of cardiovascular events in HCH patients between 33% [11] and 74% [12,13] when comparing the situation before the start of LA therapy and during the extracorporeal treatment. With such a study design, the reduction rates of CVE under LA treatment exceeded 80% in patients with elevated Lp(a) concentrations [8,11,13,14,15,16]. Specific columns against Lp(a) were shown to be able to reduce atherosclerosis at the coronaries when comparing with a statin treatment [17].

A single-blind randomized controlled trial was conducted in 20 patients with refractory angina and raised lipoprotein(a) >50 mg/dL, with 3 months of blind weekly LA or sham, followed by crossover [18,19]. The LA sessions increased myocardial perfusion, improvements with apheresis compared with sham also occurred in atherosclerotic burden, as assessed by total carotid wall volume, exercise capacity by the 6 min walk test, 4 of 5 domains of the Seattle angina questionnaire, and quality of life physical component summary according to the short form 36 (SF-36) survey.

Previously, we had shown an improvement of ocular perfusion following LA sessions [20,21].

### 5.2. Comparison of Different LA Methods

**Figure 3:**
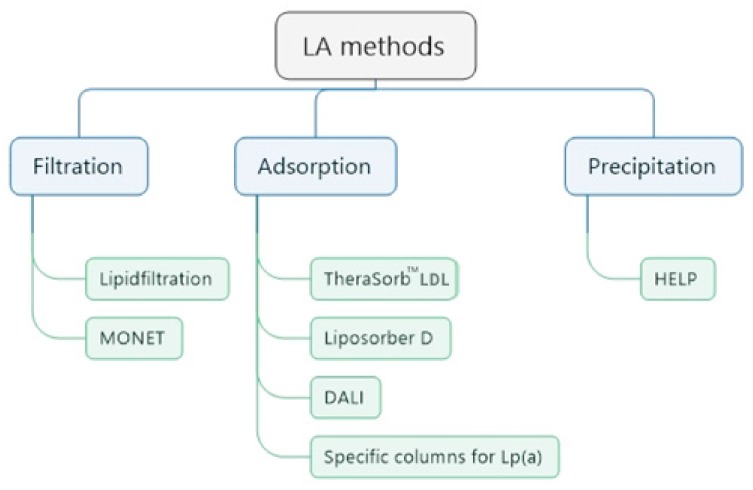
Principles of available LA methods.

The underlying principles of LA methods are filtration, adsorption, and precipitation (Figure 3) [22]. Two methods process whole blood (DALI, Liposorber D), the other methods need plasma separation as a first step.

In filtration methods, plasma is separated from the blood cells by a filter; a second filter retains macromolecules like lipoproteins. Antibodies fixed on sepharose against apolipoprotein B (TheraSorb^TM^ LDL) and against Lp(a) particles (Pocard Lp(a) columns) specifically bind the corresponding lipoproteins. An adsorption of apolipoprotein B-containing lipoproteins on a negatively charged surface takes place in the DALI and Liposorber D systems. Precipitation of lipoproteins occurs in an acidic milieu (acetate buffer) with an excess of heparin (HELP method).

Anticoagulation always has to be performed—with heparin and/or citrate.

When comparing the available LA methods with respect to their lipid-lowering capacity, only minor differences were observed [22,23]. For a given patient, the treated volume of blood/plasma has to be established by the treating physician, based on recommendations from the manufacturer. It can be modified when reduction rates for lipoproteins are too low. Otherwise, the patient has to be switched to another LA method.

Clearly, these methods have various effects on the coagulation system [23,24], on other plasma proteins and immunoglobulins [25], and on other systems [22]. The clinical significance of the pleiotropic effects for the outcome of the patients is still not fully understood. At least, an acute reduction of fibrinogen improves blood flow, and a decrease of C-reactive protein may have an anti-inflammatory effect. However, some effects are rather short-lasting (for instance on PCSK9 [26], on cytokines).

The majority of LA methods reduces both LDL-C and Lp(a). It is not possible to say what reduction is responsible for the clinical effects of LA in a given patient. The Pocard specific Lp(a) columns only decrease Lp(a)—the LDL-C concentration is hardly affected. This LA method could be preferred in patients whose LDL-C is rather low. Unfortunately, no data comparing outcome data with different LA methods have been published.

### 5.3. Adverse Effects of LA

Usually, LA methods are well tolerated. Major problems may occur with the venous access, with acute hypotension [27], and in some patients it may be necessary to start another method, for instance, when a heparin allergy appeared (such as performing another method which does not need heparin). Some patients need a calcium substitution (citrate lowers plasma calcium) or iron replacement (either orally or by infusion). The danger of bleeding should be taken into attention. ACE inhibitors should be avoided in patients on LA therapy—they may aggravate bradykinine syndrome.

Only a few patients stop LA treatment because of adverse effects.

## 6. New Drugs and LA

### 6.1. PCSK9 Inhibitors

The indication to use PCSK9 inhibitors is similar to that for LA: high LDL-C concentrations and atherosclerotic disease. Recently, outcome data with evolocumab (Fourier Study) [28,29,30,31,32] and with alirocumab (Odyssee Outcome Study; presented at American College of Cardiology—67th Scientific Sessions 10 March 2018) have been published. In both studies, the PCSK9 inhibitor decreased the incidence of CVE by 15–20% compared with placebo. Very low LDL-C levels did not induce any severe adverse effects.

**Figure 4:**
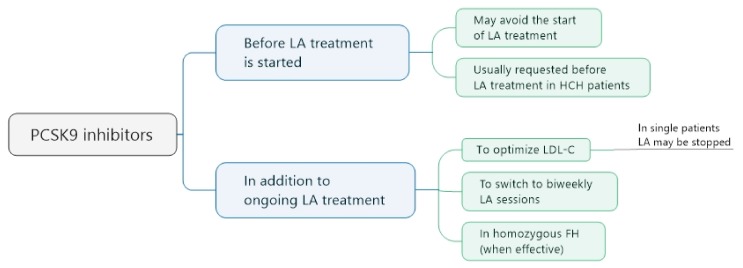
PCSK9 inhibitors and LA in HCH patients.

The trend goes in the direction that before starting LA treatment in HCH patients, a PCSK9 inhibitor has to be administered, in addition to a statin and/or other LDL-C-lowering drugs (Figure 4). In those patients who reach LDL-C levels below the target (usually 1.8 mmol/L), the extracorporeal therapy is not necessary. In patients who do not show such a reduction of LDL-C, or who do not tolerate the PCSK9 inhibitor, LA treatment can be started. An elevation of Lp(a) is not an accepted indication for PCSK9 inhibitors.

On the other hand, in patients on LA therapy, relatively high pre-LA LDL-C levels could lead to the suggestion to add a PCSK9 inhibitor to LA (Figure 4). Some of these patients can even stop the extracorporeal therapy (when very low LDL-C concentrations are measured, and the cardiovascular situation is stable). Other patients can be switched to biweekly LA sessions—a stable cardiovascular situation is a prerequisite. The injections of the PCSK9 inhibitor are usually performed after the LA sessions—it is not excluded that the drug is washed out by the extracorporeal therapy. A concomitant elevation of Lp(a) concentrations is generally regarded as a reason to continue the LA treatment in the previous way (weekly sessions).

Taking into account that a LA therapy in homozygous FH (familial HCH) patients often does not lower extremely increased LDL-C levels enough, the administration of PCSK9 inhibitors makes sense (when they are really effective).

### 6.2. Inhibitor of Microsomal Transfer Protein (MTP) in Homozygous Familial HCH

A possibility to further decrease LDL-C levels represents Lomitapide, an MTP inhibitor. It is approved by EMA (as Lojuxta) and by FDA and JFDA (as Juxtapid) for the treatment of adult patients with homozygous familial HCH. It is efficacious in these patients with mean LDL-C reductions between 50–75% [33,34], enabling patients to achieve recommended LDL-C target levels and enabling patients to discontinue concomitant apheresis [34,35]. Treatment dose needs to be titrated to balance efficacy with the tolerability of the drug related to the mechanism of action, including GI (gastro-intestinal) adverse effects and liver effects, including hepatic steatosis and transaminase elevations. Long-term studies observing efficacy and safety to 5.7 years [36] did not identify any additional safety and tolerability issues.

### 6.3. Antisense Oligonucleotide against Apolipoprotein(a)

The synthesis of apolipoprotein(a) can be effectively decreased by injections of an antisense oligonucleotide [37]. A newer formulation with a triantennary *N*-acetylgalactosamine (GalNAc3) complex did not induce any adverse effects [38]. The action of this drug lasts several weeks, and reductions of Lp(a) of up to 90% were seen. Phase III studies will soon be started. Eventually, outcome data will be required before this drug will replace LA in a large scale. However, in single cases, it may be used as an orphan drug—especially in patients who suffer from new CVE, despite an ongoing LA therapy.

## 7. Conclusions

LA represents a laborious and expensive (in Germany, costs amount to about 50,000 EUR per year per patient) therapeutic approach which should be applied after all other treatment possibilities have been used. Unfortunately, LA is accessible only in a few countries.

LA is a life-saving therapy in patients with homozygous FH in whom usual lipid-lowering drugs show a limited effectiveness. In heterozygous and polygenetic HCH, LA will keep its place only in those patients who either do not reach target levels for LDL-C or do not tolerate available lipid-lowering drugs (including PCSK9 inhibitors). In the last years, an elevation of Lp(a) appeared to be an indication for LA to improve the prognosis in high-risk patients (early CVE, multiple CVE, affection of several vascular territories). Specific anti-Lp(a) columns are a therapeutic option which need to be tested in further studies. The role of the new antisense oligonucleotide against apolipoprotein(a) will still have to be determined.

## Abbreviations

HELP	heparin-induced extracorporeal LDL precipitation
DALI	direct adsorption of lipoproteins
MONET	membrane filtration optimized novel extracorporeal treatment
RCT	randomized control trial

## References

[1] Julius U. (2017). History of lipidology and lipoprotein apheresis. Atheroscler. Suppl..

[2] Cannon C.P., Blazing M.A., Giugliano R.P., McCagg A., White J.A., Theroux P., Darius H., Lewis B.S., Ophuis T.O., Jukema J.W. (2015). Ezetimibe added to statin therapy after acute coronary syndromes. N. Engl. J. Med..

[3] Thompson G.R., Catapano A., Saheb S., Atassi-Dumont M., Barbir M., Eriksson M., Paulweber B., Sijbrands E., Stalenhoef A.F., Parhofer K.G. (2010). Severe hypercholesterolaemia: Therapeutic goals and eligibility criteria for LDL apheresis in Europe. Curr. Opin. Lipidol..

[4] Thompson G.R., Seed M., Naoumova R.P., Neuwirth C., Walji S., Aitman T.J., Scott J., Myant N.B., Soutar A.K. (2015). Improved cardiovascular outcomes following temporal advances in lipid-lowering therapy in a genetically-characterised cohort of familial hypercholesterolaemia homozygotes. Atherosclerosis.

[B5-jcdd-05-00027] 5.Bundesministerium für Gesundheit, Bekanntmachung des Bundesausschusses der Ärzte und Krankenkassen über eine Änderung der Richtlinien über die Bewertung ärztlicher Untersuchungs- und Behandlungsmethoden gemäß § 135 Abs. 1 des Fünften Buches Sozialgesetzbuch (SGB V) (BUB-Richtlinien), BAnz, **2003**; 123:14486.

[B6-jcdd-05-00027] 6.Bundesministerium für Gesundheit, Bekanntmachung eines Beschlusses des Gemeinsamen Bundesausschusses über eine Änderung der Richtlinie Methoden vertragsärztliche Versorgung: Apherese bei isolierter Lp(a)-Erhöhung, BAnz, **2008**; 138:3321.

[7] Julius U. (2015). Niacin as antidyslipidemic drug. Can. J. Physiol. Pharmacol..

[8] Leebmann J., Roeseler E., Julius U., Heigl F., Spitthoever R., Heutling D., Breitenberger P., Maerz W., Lehmacher W., Heibges A. (2013). Lipoprotein apheresis in patients with maximally tolerated lipid-lowering therapy, lipoprotein(a)-hyperlipoproteinemia, and progressive cardiovascular disease: Prospective observational multicenter study. Circulation.

[9] Gross E., Hohenstein B., Julius U. (2015). Effects of lipoprotein apheresis on the lipoprotein(a) levels in the long run. Atheroscler. Suppl..

[10] Mabuchi H., Koizumi J., Shimizu M., Kajinami K., Miyamoto S., Ueda K., Takegoshi T. (1998). Long-term efficacy of low-density lipoprotein apheresis on coronary heart disease in familial hypercholesterolemia. Hokuriku-FH-LDL-apheresis study group. Am. J. Cardiol..

[11] Emmrich U., Hohenstein B., Julius U. (2015). Actual situation of lipoprotein apheresis in Saxony in 2013. Atheroscler. Suppl..

[12] Heigl F., Hettich R., Lotz N., Reeg H., Pflederer T., Osterkorn D., Osterkorn K., Klingel R. (2015). Efficacy, safety, and tolerability of long-term lipoprotein apheresis in patients with LDL- or Lp(a) hyperlipoproteinemia: Findings gathered from more than 36,000 treatments at one center in Germany. Atheroscler. Suppl..

[13] Schatz U., Tselmin S., Muller G., Julius U., Hohenstein B., Fischer S., Bornstein S.R. (2017). Most significant reduction of cardiovascular events in patients undergoing lipoproteinapheresis due to raised Lp(a) levels—A multicenter observational study. Atheroscler. Suppl..

[14] Von Dryander M., Fischer S., Passauer J., Muller G., Bornstein S.R., Julius U. (2013). Differences in the atherogenic risk of patients treated by lipoprotein apheresis according to their lipid pattern. Atheroscler. Suppl..

[15] Jaeger B.R., Richter Y., Nagel D., Heigl F., Vogt A., Roeseler E., Parhofer K., Ramlow W., Koch M., Utermann G. (2009). Longitudinal cohort study on the effectiveness of lipid apheresis treatment to reduce high lipoprotein(a) levels and prevent major adverse coronary events. Nat. Clin. Pract. Cardiovasc. Med..

[16] Roeseler E., Julius U., Heigl F., Spitthoever R., Heutling D., Breitenberger P., Leebmann J., Lehmacher W., Kamstrup P.R., Nordestgaard B.G. (2016). Lipoprotein apheresis for lipoprotein(a)-associated cardiovascular disease: Prospective 5 years of follow-up and apo(a) characterization. Arterioscler. Thromb. Vasc. Biol..

[17] Safarova M.S., Ezhov M.V., Afanasieva O.I., Matchin Y.G., Atanesyan R.V., Adamova I.Y., Utkina E.A., Konovalov G.A., Pokrovsky S.N. (2013). Effect of specific lipoprotein(a) apheresis on coronary atherosclerosis regression assessed by quantitative coronary angiography. Atheroscler. Suppl..

[18] Khan T.Z., Hsu L.Y., Arai A.E., Rhodes S., Pottle A., Wage R., Banya W., Gatehouse P.D., Giri S., Collins P. (2017). Apheresis as novel treatment for refractory angina with raised lipoprotein(a): A randomized controlled cross-over trial. Eur. Heart J..

[19] Khan T.Z. (2018). Can lipoprotein apheresis offer a therapeutic role in the management of patients with refractory angina and raised lipoprotein(a)?. Ther. Apher. Dial..

[20] Reimann M., Prieur S., Lippold B., Bornstein S.R., Reichmann H., Julius U., Ziemssen T. (2009). Retinal vessel analysis in hypercholesterolemic patients before and after LDL apheresis. Atheroscler. Suppl..

[21] Terai N., Julius U., Haustein M., Spoerl E., Pillunat L.E. (2011). The effect of low-density lipoprotein apheresis on ocular microcirculation in patients with hypercholesterolaemia: A pilot study. Br. J. Ophthalmol..

[22] Julius U. (2016). Lipoprotein apheresis in the management of severe hypercholesterolemia and of elevation of lipoprotein(a): Current perspectives and patient selection. Med. Devices.

[23] Julius U., Fischer S., Schatz U., Passauer J., Bornstein S. (2013). Why an apheresis center should offer more than one lipoprotein apheresis method. Ther. Apher. Dial..

[24] Julius U., Metzler W., Pietzsch J., Fassbender T., Klingel R. (2002). Intraindividual comparison of two extracorporeal LDL apheresis methods: Lipidfiltration and HELP. Int. J. Artif. Organs.

[25] Julius U., Siegert G., Kostka H., Schatz U., Hohenstein B. (2015). Effects of different lipoprotein apheresis methods on serum protein levels. Atheroscler. Suppl..

[26] Julius U., Milton M., Stoellner D., Rader D., Gordon B., Polk D., Waldmann E., Parhofer K.G., Moriarty P.M. (2015). Effects of lipoprotein apheresis on PCSK9 levels. Atheroscler. Suppl..

[27] Dittrich-Riediger J., Schatz U., Hohenstein B., Julius U. (2015). Adverse events of lipoprotein apheresis and immunoadsorption at the apheresis center at the University Hospital Dresden. Atheroscler. Suppl..

[28] Sabatine M.S., Leiter L.A., Wiviott S.D., Giugliano R.P., Deedwania P., De Ferrari G.M., Murphy S.A., Kuder J.F., Gouni-Berthold I., Lewis B.S. (2017). Cardiovascular safety and efficacy of the PCSK9 inhibitor evolocumab in patients with and without diabetes and the effect of evolocumab on glycaemia and risk of new-onset diabetes: A prespecified analysis of the Fourier randomised controlled trial. Lancet Diabetes Endocrinol..

[29] Bonaca M.P., Nault P., Giugliano R.P., Keech A.C., Pineda A.L., Kanevsky E., Kuder J., Murphy S.A., Jukema J.W., Lewis B.S. (2018). Low-density lipoprotein cholesterol lowering with evolocumab and outcomes in patients with peripheral artery disease: Insights from the Fourier trial (further cardiovascular outcomes research with PCSK9 inhibition in subjects with elevated risk). Circulation.

[30] Giugliano R.P., Pedersen T.R., Park J.G., De Ferrari G.M., Gaciong Z.A., Ceska R., Toth K., Gouni-Berthold I., Lopez-Miranda J., Schiele F. (2017). Clinical efficacy and safety of achieving very low LDL-cholesterol concentrations with the PCSK9 inhibitor evolocumab: A prespecified secondary analysis of the Fourier trial. Lancet.

[31] Giugliano R.P., Mach F., Zavitz K., Kurtz C., Schneider J., Wang H., Keech A., Pedersen T.R., Sabatine M.S., Sever P.S. (2017). Design and rationale of the Ebbinghaus trial: A phase 3, double-blind, placebo-controlled, multicenter study to assess the effect of evolocumab on cognitive function in patients with clinically evident cardiovascular disease and receiving statin background lipid-lowering therapy-a cognitive study of patients enrolled in the Fourier trial. Clin. Cardiol..

[32] Sabatine M.S., Giugliano R.P., Keech A.C., Honarpour N., Wiviott S.D., Murphy S.A., Kuder J.F., Wang H., Liu T., Wasserman S.M. (2017). Evolocumab and clinical outcomes in patients with cardiovascular disease. N. Engl. J. Med..

[33] Cuchel M., Meagher E.A., du Toit T.H., Blom D.J., Marais A.D., Hegele R.A., Averna M.R., Sirtori C.R., Shah P.K., Gaudet D. (2013). Efficacy and safety of a microsomal triglyceride transfer protein inhibitor in patients with homozygous familial hypercholesterolaemia: A single-arm, open-label, phase 3 study. Lancet.

[34] D’Erasmo L., Cefalu A.B., Noto D., Giammanco A., Averna M., Pintus P., Medde P., Vigna G.B., Sirtori C., Calabresi L. (2017). Efficacy of lomitapide in the treatment of familial homozygous hypercholesterolemia: Results of a real-world clinical experience in Italy. Adv. Ther..

[35] Cuchel M., Bruckert E., Ginsberg H.N., Raal F.J., Santos R.D., Hegele R.A., Kuivenhoven J.A., Nordestgaard B.G., Descamps O.S., Steinhagen-Thiessen E. (2014). Homozygous familial hypercholesterolaemia: New insights and guidance for clinicians to improve detection and clinical management. A position paper from the consensus panel on familial hypercholesterolaemia of the European Atherosclerosis Society. Eur. Heart J..

[36] Blom D.J., Averna M.R., Meagher E.A., du Toit Theron H., Sirtori C.R., Hegele R.A., Shah P.K., Gaudet D., Stefanutti C., Vigna G.B. (2017). Long-term efficacy and safety of the microsomal triglyceride transfer protein inhibitor lomitapide in patients with homozygous familial hypercholesterolemia. Circulation.

[37] Tsimikas S., Viney N.J., Hughes S.G., Singleton W., Graham M.J., Baker B.F., Burkey J.L., Yang Q., Marcovina S.M., Geary R.S. (2015). Antisense therapy targeting apolipoprotein(a): A randomised, double-blind, placebo-controlled phase 1 study. Lancet.

[38] Viney N.J., van Capelleveen J.C., Geary R.S., Xia S., Tami J.A., Yu R.Z., Marcovina S.M., Hughes S.G., Graham M.J., Crooke R.M. (2016). Antisense oligonucleotides targeting apolipoprotein(a) in people with raised lipoprotein(a): Two randomised, double-blind, placebo-controlled, dose-ranging trials. Lancet.

